# Test for Prussic Acid

**Published:** 1829

**Authors:** 


34. *Test for Prussic Acid.*—Dr. Edward Turner, Professor of Chemistry, in the new London University, in a letter to the Editors of the Edinburgh Medical and Surgical Journal, for October, 1828, after considering the relative value of the nitrate of silver, the sulphate of copper, the per-sulphate of iron and the proto-sulphate of the same metal, gives a decided preference to the last; as may be seen by the following extract:—

The proper test of prussic acid is the common proto-sulphate of iron, the green vitriol of commerce. If recently prepared, the precipitate left after dissolving the excess of oxide will at first be green, and become blue after exposure for a few minutes to the air. But if the solution has been previously kept for some hours in an open vessel, so that it may contain both oxides, perfect Prussian blue will be formed at once. The proto-sulphate of iron, even in cases of judicial inquiry, is the only chemical re-agent required. M. Lassaigne recommends the sulphate of copper as a re-agent twice as delicate as per-sulphate of iron—an opinion which is quite correct. But it is considerably less delicate than the proto-sulphate, and besides, the white tint of the cyanuret of copper is far less characteristic than the rare and well marked color of Prussian blue. Sulphate of copper, as a test of prussic acid, is therefore unnecessary. These remarks render it probable, that, in animals killed by prussic acid, the poison may be detected at longer periods after death than was observed in the experiments of M M. Leuret and Lassaigne.

